# Immunogenicity and protection of a *Pasteurella multocida* strain with a truncated lipopolysaccharide outer core in ducks

**DOI:** 10.1186/s13567-022-01035-y

**Published:** 2022-03-02

**Authors:** Xinxin Zhao, Fuxiang Yang, Hui Shen, Yi Liao, Dekang Zhu, Mingshu Wang, Renyong Jia, Shun Chen, Mafeng Liu, Qiao Yang, Ying Wu, Shaqiu Zhang, Juan Huang, Xumin Ou, Sai Mao, Qun Gao, Di Sun, Bin Tian, Anchun Cheng

**Affiliations:** 1 Research Center of Avian Diseases, College of Veterinary Medicine, Sichuan Agricultural University, Chengdu, 611130 Sichuan China; 2 Institute of Preventive Veterinary Medicine, Sichuan Agricultural University, Chengdu, 611130 Sichuan China; 3 Key Laboratory of Animal Disease and Human Health of Sichuan Province, Chengdu, 611130 Sichuan China

**Keywords:** *Pasteurella multocida*, LPS, *gatA*, *hptE*, virulence, live attenuated vaccine

## Abstract

*Pasteurella multocida* infection frequently causes fowl cholera outbreaks, leading to huge economic losses to the poultry industry worldwide. This study developed a novel live attenuated *P. multocida* vaccine strain for ducks named PMZ2 with deletion of the *gatA* gene and first four bases of the *hptE* gene, both of which are required for the synthesis of the lipopolysaccharide (LPS) outer core. PMZ2 produced a truncated LPS phenotype and was highly attenuated in ducks with a > 10^5^-fold higher LD_50_ than the wild-type strain. PMZ2 colonized the blood and organs, including the spleen, liver and lung, at remarkably reduced levels, and its high dose of oral infection did not cause adverse effects on body temperatures and body weights in ducks. To evaluate the vaccine efficacy of the mutant, ducklings were inoculated orally or intranasally with PMZ2 or PBS twice and subsequently subjected to a lethal challenge. Compared with the PBS control, PMZ2 immunization stimulated significantly elevated serum IgG, bile IgA and tracheal IgA responses, especially after the boost immunization in both the oral and intranasal groups, and the induced serum had significant bactericidal effects against the wild-type strain. Furthermore, the two PMZ2 immunization groups exhibited alleviated tissue lesions and significantly decreased bacterial loads in the blood and organs compared with the PBS group post-challenge. All the ducks in the PMZ2 oral and intranasal groups survived the challenge, while 70% of ducks in the PBS group succumbed to the challenge. Thus, the *P. multocida* mutant with mutation of the *gatA* gene and part of the *hptE* gene proved to be an effective live attenuated vaccine candidate for prevention of fowl cholera in ducks.

**Supplementary Information:**

The online version contains supplementary material available at 10.1186/s13567-022-01035-y.

## Introduction

*Pasteurella multocida* (*P. multocida*) is an important zoonotic bacterium that can cause a variety of severe diseases in economically valuable farm animals. It manifests as fowl cholera (FC) in avian species, leading to significant economic losses in the poultry industry due to acute or peracute cases with signs of systemic disease [1]. Infection with virulent *P. multocida* strains usually induces gross lesions in livers, spleens and other viscera and results in animal deaths in several days. *P. multocida* isolates are classified into five capsular serogroups (A, B, D, E, F) and 16 Heddleston lipopolysaccharide (LPS) serovars [2]. They can currently also be divided into 8 LPS genotypes (L1-L8) according to the LPS outer core gene cluster [3]. FC is caused largely by strains belonging to capsular type A or Heddleston serovar 1, 3 and 4 [4, 5]. Antibiotics are the main means for the treatment of pasteurellosis in animals, but they are practically useless in peracute to acute pasteurellosis such as haemorrhagic septicaemia. Also, this strategy is expensive and lengthy and becomes ineffective because of the increased drug resistance of the bacterium [6]. Additionally, excessive use of antibiotics can cause toxicity to human consumers [7]. These drawbacks highlight the importance of effective vaccines that are the most economic and potent tool to prevent such infectious diseases.

The formalin-killed inactivated bacterins and the naturally occurring live Clemson University (CU) vaccine strain that are the most widely used have been licenced for FC prevention. Immunization with bacterins stimulates protective immunity against homologous challenge in poultry [8]; however, they must be injected and lack the ability to induce cross serotype protection and may cause adverse effects in the injection sites [9]. The CU vaccine can incur specific cellular and humoural responses in the respiratory organs and provide long-term protection in turkeys without obvious adverse effects. It was proven that the CU vaccine offered stronger overall protection than oil-based bacterins in broiler chickens [10]. Nevertheless, turkeys that had been vaccinated with the CU vaccine subsequently still experienced outbreaks of fowl cholera [11, 12], and the CU vaccine under certain conditions still causes clinical disease because of reversion to virulence [13]. Thus, these limitations necessitate developing novel vaccines with sufficient safety and protection against FC. Considering the advantages of live attenuated vaccines, including the ability to stimulate cross-serotype protection, ease of preparation and needle-free administration [14], exploration of novel live attenuated vaccines is attractive and should be given priority for constructing a new generation of *P. multocida* vaccines.

With the development of genetic technology in bacteria, deletion of the gene affecting bacterial virulence or metabolism has become a powerful approach for the construction of rationally attenuated strains. Site-directed gene mutation is of high efficiency and clarity for genetic modification [15]. Mutation of the *aroA* gene required for the synthesis of aromatic amino acids in the *P. multocida* A:1 or A:3 strain led to a striking reduction in virulence, and auxotrophic derivatives were capable of providing both homologous and heterologous protection against wild-type (WT) challenge in chickens [16, 17]. Later, an acapsular *P. multocida* A:1 strain with deletion of the *hexA* gene was constructed and proven to be attenuated in virulence and could induce a high level of protection against lethal challenge in chickens [18]. Moreover, live attenuated *aroA* derivative and *gdhA* derivative of *Pasteurella multocida* B:2 are effective to protect calves and buffaloes against hemorrhagic septicaemia, respectively [19, 20]. Thus, mutating a certain virulence gene is a good strategy for constructing live attenuated vaccines of *P. multocida*; however, no such vaccines have been explored for ducks.

The *P. multocida* LPS does not contain O-antigen and consists of three distinct regions: a hydrophobic lipid A domain, an inner core oligosaccharide and an outer core oligosaccharide. The outer core is diverse among Heddleston serovars, and its assembly in the genotype L1 strain is dependent on six genes. The transferase genes *htpE* and *gatA* are responsible for adding Heptose (Hep) IV to the inner core and transferring both Galactose (Gal) I and Gal II to Hep IV, respectively. The remaining genes *pcgA*, *pcgB*, *pcgC* and *pcgD* work together in the synthesis of phosphocholine, transferring it to both Gal residues. Inactivation of the *hptE* or *gatA* gene of genotype L1 strain VP161 caused reduced bacterial loads in tissues and virulence attenuation in chickens [21], while mutation of *pcgC* had little effect on bacterial virulence [22]. Nevertheless, our study recently demonstrated that deletion of *pcgD* of the genotype L1 strain PM0818 results in significant and greater virulence reduction than *hptE* mutation in ducks upon either oral or intramuscular challenge [23]. These studies suggest that the LPS outer core is factually important for the pathogenesis of the genotype L1 strain in poultry, while the role of each synthesis gene of the outer core in bacterial virulence is very likely dissimilar in chickens and ducks.

As the *pcgD* mutant of *P. multocida* can cause death of ducks at a high dose of oral infection [23], genes that have greater impacts on virulence still need to be searched for. In this study, we constructed a *P. multocida* mutant named PMZ2 (ΔgatA^*^) with deletion of the entire *gatA* gene and first four nucleotide bases (ATGC) of the *hptE* coding sequence in the wild-type (WT) PM0818 strain via suicide plasmid-mediated homologous recombination. We originally attempted to construct the *gatA* mutant to study its role in virulence; however, due to an incorrect primer design, the resulting mutation also disrupted the starting codon of the *hptE* gene. Interestingly, PMZ2 (ΔgatA^*^) was found to be less virulent than the *pcgD* mutant and highly attenuated with a 10^5^-fold higher median lethal dose (LD_50_) than the WT strain. Due to the sharp reduction in virulence, we aimed to evaluate the vaccine potential of PMZ2 in terms of safety, immunogenicity and protection efficacy in ducks.

## Materials and methods

### Bacterial strains and growth conditions

The bacterial strains and plasmids used are listed in Table 1. The mutant strain was constructed based on the *P. multocida* WT strain PM0818 belonging to LPS genotype L1 [23]. *P. multocida* strains were cultured at 37 °C in brain heart infusion (BHI) broth or on BHI agar (BD Bioscience, USA), and *Escherichia coli* (*E. coli*) strains were grown in Luria–Bertani (LB) broth or on LB agar (Coolaber, Beijing, China). Tryptic soybean agar (TSA, Difco Laboratories, USA) was generally used for colony counts of *P. multocida* strains. When required, antibiotics were added to the medium at the following concentrations: kanamycin, 50 μg/mL and chloramphenicol, 25 μg/mL.

**Table 1:** **Bacterial strains and plasmids used in this study**

Strains or plasmids	Description	Source
Plasmids		
pMC-Express	A broad host-range shuttle vector derived from pMIDG100, *sodC* promoter, Cm^r^	[26]
pCZb5	pMC-Express derivative with a 250 bp fragment containing the *tpiA* promoter	This work
pRE112	*sacB* mobRP4 R6K ori Cm^r^	[24]
pCZ52	pRE112-ΔgatA^*^	This work
pCZ57	Insertion of complete *gatA* into pCZb5	This work
pCZ58	Insertion of complete *gatA* and *hptE* into pCZb5	This work
Strains		
SM10 *λ pir*	*E. coli thi thr*-1 *leu*6 *pro*A2 *his*-4 *arg* E2 *lac*Y1 *galK*2*, ara*14*xy*l5 *supE*44*, λpir*	[25]
PM0818	*P. multocida* 0818, Wild-type and virulent, LPS genotype L1	[23]
PMZ2	PM0818 Δ*gatA*::*kanR*	This work

### Plasmid and mutant strain construction

The primers used in this study are listed in Additional file 1. The *P. multocida* mutant strain PMZ2 was constructed by allelic exchange using the suicide T-vector pRE112 [24] as previously described [23]. In brief, the upstream segment (457 bp) and downstream segment (493 bp) of the gatA^*^ gene were cloned with primer pairs DgatA^*^-1F/1R and DgatA^*^-2F/2R from the PM0818 genome, respectively, and then the two sequences were linked with the kanamycin resistance (*kanR*) gene (853 bp) amplified with *kanR*-gatA^*^-F/R from the pET28a plasmid by overlap PCR. The resulting PCR product was ligated into pRE112 by double-enzyme digestion with KpnI and XmaI generating the plasmid pCZ52, which carried a deletion of the entire *gatA* gene sequence and first four bases (ATGC) of the *hptE* gene. This plasmid was subsequently transformed into the *P. multocida* WT strain from *E. coli* SM10 λ pir [25] via conjugation, and the mutant strain designated PMZ2 was selected on BHI agar containing kanamycin. The target gene mutation was verified by PCR with three primer pairs, MIA-1F/P1-R, P2-F/MIA-2R and MIA-3F/3R, for the deleted region and flanking DNA, and the PCR product amplified from PMZ2 with MIA-1F/2R was subjected to DNA sequencing (BGI-Shenzhen, China). The primer sequences of P1-R and P2-F have been provided previously [23].

To complement gene mutation in PMZ2, the *sodC* promoter of pMC-Express [26] was replaced with the *tpiA* promoter (250 bp upstream of the start codon) amplified by the primer pair *tpiA*-F/R through double-enzyme digestion with XhoI and EcoRI, generating the modified pMC-Express named pCZb5. Then, the *gatA* sequence or the *gatA* and *hptE* sequence was amplified from the PM0818 genome with the primer pairs C*gatA*-F/R and C*gatA-hptE*-F/R, respectively, and ligated into the KpnI and NotI sites of pCZb5 to generate the complementary plasmid pCZ57 or pCZ58. Finally, the two recombinant plasmids were transformed into PMZ2, generating two complemented strains, PMZ2 (pCZ57-*gatA*) and PMZ2 (pCZ58-*gatA-hptE*), respectively.

### Detection of LPS phenotypes

The LPS phenotypes of the *P. multocida* strains were visualized by silver staining as previously described [23, 27]. In brief, whole-cell lysates of the *P. multocida* WT strain or mutant train were subjected to sodium dodecyl sulphate–polyacrylamide gel electrophoresis (SDS-PAGE) on 15% (w/v) acrylamide gels using a Tricine-SDS buffer system (Bio-Rad Laboratories, California, USA). Then, the gels were stained with silver solution (4% silver nitrate, 46.4% sodium hydroxide and 0.8% ammonia water) followed by incubation with developing solution (0.05% sodium citrate, 0.5% formaldehyde) and ultrapure water successively.

### Virulence and bacterial concentration of the *P. multocida* strains in ducks

For comparison of the virulence of PMZ1 (Δ*pcgD*) and PMZ2 (ΔgatA^*^), 7-day-old ducks (*n* = 10/group) purchased from Grimaud Breeding Co., Ltd. (Chengdu, China) were inoculated with 10^9^ colony forming units (CFU) of PMZ1 or PMZ2 orally, and then the animal mortality of each group was determined with a 7-day period. To determine the LD_50_ of the bacterial strains, tenfold serial dilutions of the CFU of the WT strain or PMZ2 strain were orally or intramuscularly inoculated into groups of 7-day-old ducklings (*n* = 6 per dose). Animal survival was observed over a period of 14 days after infection. The LD_50_ was calculated using the method of Reed and Muench. Additionally, for determination of bacterial concentration, ducklings (*n* = 6 per group) were orally inoculated with approximately 10^9^ CFU of the WT strain or PMZ2 strain, and blood and organs, including the spleen, liver and lungs, were sampled 24 h post-infection. Next, the bacterial concentrations were calculated as CFU per mL of blood (CFU/mL) or CFU per gram of tissue as previously described [23].

### Safety assay

To observe whether PMZ2 infection could cause adverse effects on animal growth, seven-day-old ducklings (*n* = 10/group) were inoculated with PBS or 10^9^ CFU of the PMZ2 strain orally, and then the clinical signs, rectal temperature and body weights of the ducks were recorded every two days within a period of 14 days.

### Immunization and challenge

The vaccine strain PMZ2 (ΔgatA^*^) was grown statically overnight at 37 °C. On the second day, the growth culture was diluted 1:100 in fresh BHI medium, and the bacteria were grown continually to an OD_600_ of 0.6–0.8. Then, the bacteria were diluted with PBS. Groups of 7-day-old ducks (*n* = 24 per group) were orally or intranasally immunized with approximately 10^8^ CFU of PMZ2 and then revaccinated 14 days later. An equal number of ducklings were also inoculated orally or intranasally with PBS at the same time as the negative control. The blood, bile and tracheas were collected from 5 randomly selected ducks of each group 7 days post-immunization for antibody detection. Sera were obtained from the blood by centrifugation at 1700 × *g* for 10 min at 4 °C. Bile collected from the gall bladder, was centrifuged at 12 000 × *g* for 5 min at 4 °C to acquire the supernatant for the analysis of IgA. The trachea segments approximately 2 cm in length were rinsed with 1 mL PBS and then centrifugated at 12 000 × *g* for 5 min at 4 °C to acquire the supernatant for the analysis of IgA. Next, all immunized ducks were challenged intramuscularly with 100-fold LD_50_ of the WT strain PM0818 14 days after the second immunization. Samples including the blood, spleen, liver and lungs were collected from 4 ducks of each group at 24 h post-challenge for the measurement of bacterial loads and pathological lesions. Finally, the survival of the remaining challenged ducks (10 ducks per group) was monitored and recorded daily for 14 days.

### Enzyme-linked immunosorbent assay (ELISA)

The antibody responses to inactivated *P. multocida* antigens were measured by indirect ELISA as previously indicated [28]. In brief, 1 × 10^9^ CFU of heating-inactivated *P. multocida* 0818 suspended in 1 mL carbonate bicarbonate buffer was added to wells of a 96-well ELISA microtiter plate for antigen coating, which was incubated at 4 °C overnight. After washing with PBS containing 0.05% Tween 20 (Amresco, USA), the plate was blocked with 5% bovine serum albumin (BSA, BD, San Diego, CA, USA) in PBS for 2 h at 37 °C. Then, 1:1000 diluted serum or 1:4 diluted bile or tracheal fluids suspended in PBS containing 1% BSA were added to the plate wells for binding. After 1 h of incubation at 37 °C and washing, the plate was further incubated with 1:10 000 diluted horseradish peroxidase (HRP)-conjugated anti-duck IgG (KPL, USA) or 1:250 diluted HRP-conjugated anti-duck IgA (Bio-Rad Laboratories, USA) for 1 h at 37 °C. After thorough washing, TMB substrate solution (Macgene, Beijing, China) was added for colouration for 10 min at room temperature, and the reaction was stopped by the addition of 2 M sulfuric acid. Finally, the optical density value at 450 nm was read using a microplate reader (Bio-Rad Laboratories).

### Serum bactericidal assay

The serum collected from ducks of each immunized group at day 21 or day 28 post-first immunization was pooled for the serum bactericidal assay as described previously [29]. The serum samples were inactivated by heating at 56 °C for 30 min. The *P. multocida* PM0818 strain was grown in BHI medium to log phase and diluted in PBS buffer to approximately 2 × 10^3^ CFU/mL. The bacteria were incubated with 90% heat-inactivated serum or active serum for 5 h at 37 °C. The relative survival was calculated as the percent CFU counted in the active serum group compared to the CFU of the inactivated serum group. Each sample and control were tested in triplicate.

### Histopathological lesions post-challenge

Detection of the histopathological changes in ducks was carried out as previously described [23]. The spleens and livers of each immunized group were fixed in 4% paraformaldehyde, dehydrated, embedded in paraffin, sectioned into 4-μm-thick sections, placed on microscope slides, and stained with haematoxylin and eosin (HE) using standard procedures. Additionally, to better observe the pathological changes, 4 ducks without immunization and challenge were included in the HE assay as the blank control.

### Statistical analysis

The data are shown as the means ± SD and were analysed by two tailed student *t* test or one-way ANOVA followed by Tukey multiple comparison test in GraphPad Prism (GraphPad Software, California, USA). The survival curves in the virulence assay were analysed by the Log-rank test. A probability value of *p* < 0.05 was considered statistically significant.

## Results

### Construction and identification of the *P. multocida* mutant

The DNA segment containing the *gatA* gene and first four bases of the *hptE* gene were deleted and replaced with the *kanR* gene in the *P. multocida* WT strain PM0818, generating the mutant designated PMZ2 (ΔgatA^*^). The DNA mutation was identified by PCR. The DNA section including the upstream sequence of *gatA* and a partial *kanR* (MIA-1F/P1-R) and the section including the downstream sequence of *gatA* and a partial *kanR* (P2-F/MIA-2R) were amplified from the PMZ2 strain but not from the WT strain, while the partial sequence of *gatA* (MIA-3F/3R) was only present in the WT strain (Additional file 2). Additionally, the DNA segment containing the flanking region of *kanR* was cloned from PMZ2 with the primers MIA-1F/2R and sequenced to confirm the exact deletion of the target sequence.

**Figure 1:**
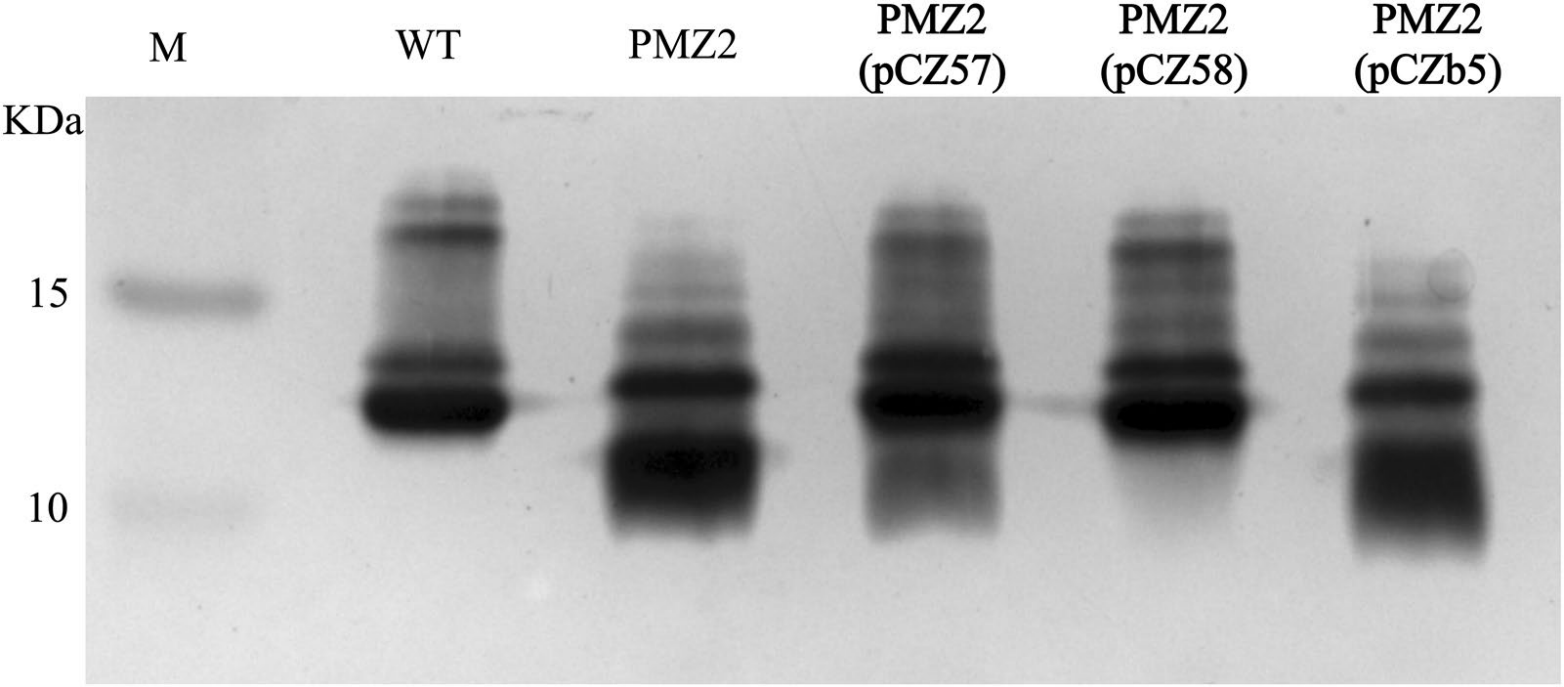
**LPS phenotypes.** LPS extracted from the *P. multocida* WT strain, the mutant strain PMZ2 (ΔgatA^*^), two complemented strains, PMZ2 (pCZ57) and PMZ2 (pCZ58), and the control strain PMZ2 (pCZb5), were subjected to sodium dodecyl sulphate–polyacrylamide gel electrophoresis followed by silver staining. M refers to the protein marker.

To further validate the PMZ2 mutant, two complemented strains, PMZ2 (pCZ57-*gatA*) and PMZ2 (pCZ58-*gatA-hptE*), were constructed, and LPS profiles of the *P. multocida* strains were detected by silver staining. Compared to a complete LPS phenotype produced by the WT strain, PMZ2 gave rise to a truncated LPS that migrated further within the gel than the WT LPS (Figure 1). Complementation of the mutation with both *gatA* and *hptE* genes in *trans* completely restored the WT LPS phenotype, whereas complementation with only the *gatA* gene achieved partial restoration as the production of a mixture of the WT and truncated LPS profiles. In contrast, PMZ2 harbouring the empty plasmid also generated a truncated LPS profile (Figure 1). Thus, the mutant strain PMZ2 lacked the *gatA* gene and possessed the *hptE* gene with functional defects.

### The virulence of the *P. multocida* mutant

**Figure 2:**
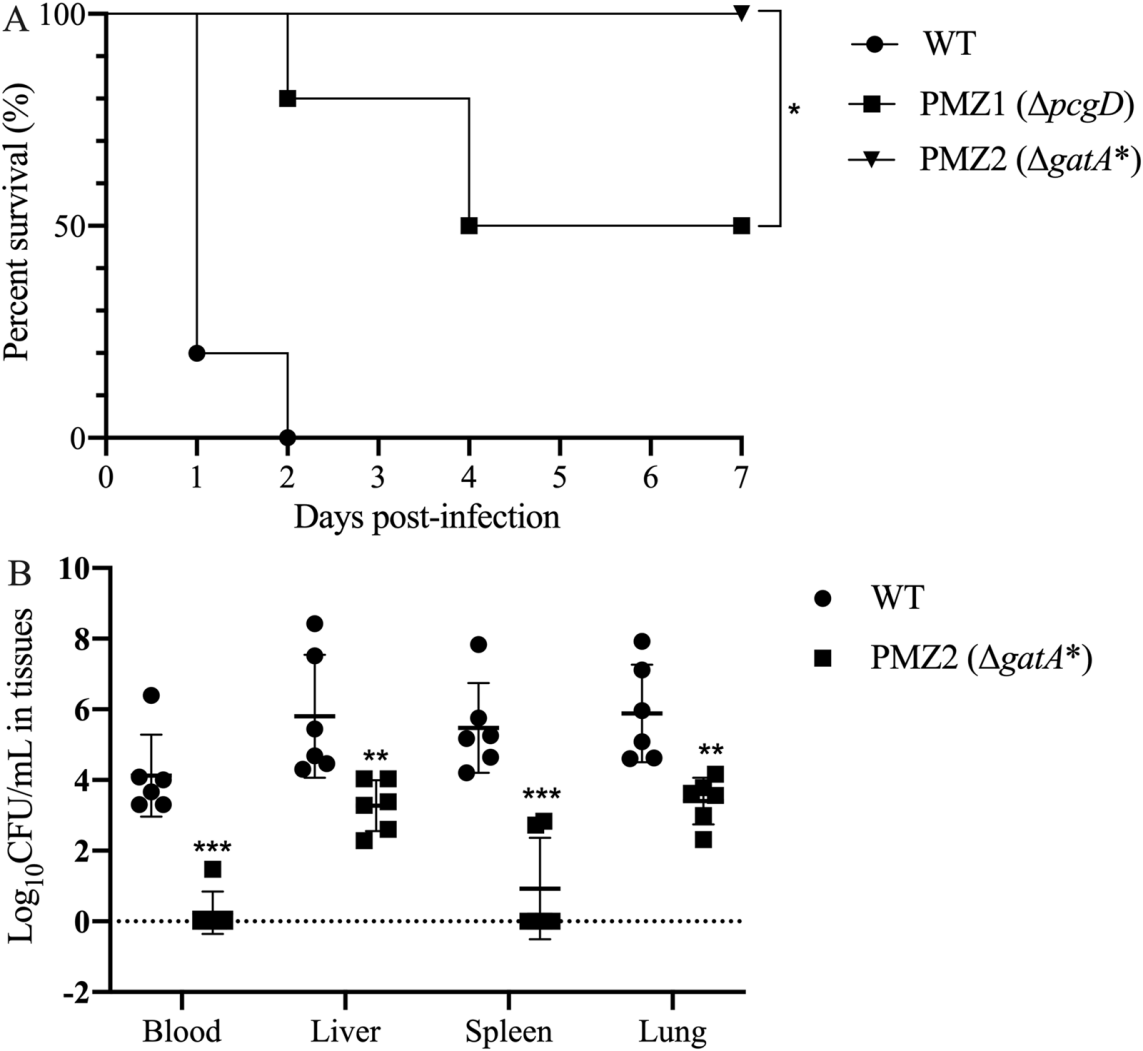
**The virulence and bacterial concentration of *P. multocida* strains.** Ducklings were inoculated orally with approximately 10^9^ CFU of the WT strain and two mutant strains, PMZ1 (Δ*pcgD*) and PMZ2 (ΔgatA^*^). Animal survival (*n* = 10/group) was monitored within 7 days post-infection (**A**), and the bacterial loads of the WT strain and PMZ2 strain (*n* = 6/group) in the blood, liver, spleen and lung were determined as CFU/mL or CFU/g at 24 h post-infection (**B**). The asterisks indicate significance among groups. *p < 0.05; **p < 0.01; ***p < 0.001.

All animals succumbed to the challenge in the WT group, whereas all animals survived infection with PMZ2, and half of the animals survived infection with PMZ1 (Figure 2A). PMZ2 was significantly less virulent than PMZ1 (log-rank test, *p* < 0.05). The bacterial counts of PMZ2 was lower in the blood, spleen, liver and lungs compared to those of the WT strain at 24 h post-high dose of oral infection (Figure 2B. Blood and spleen, *p* < 0.001; liver and lung, *p* < 0.01). In particular, PMZ2 was not isolated in the blood in five of six ducklings. Moreover, the oral LD_50_ of PMZ2 was more than 3.05 × 10^9^ CFU, which was > 10^5^-fold higher than that of the WT strain (9.63 × 10^3^ CFU); the intramuscular LD_50_ was 1.54 × 10^6^ CFU for PMZ2, also 10^5^-fold higher than that of the WT strain (14.6 CFU) (Table 2).

**Table 2:** **Determination of the LD**_**50**_ **of the** ***P. multocida*** **WT and PMZ2 strains**

Route	Strains	Challenge dose (CFU) and survival									LD_50_ (CFU)
		10	10^2^	10^3^	10^4^	10^5^	10^6^	10^7^	10^8^	10^9^	
Intramuscular	WT	3/6	1/6	0/6	0/6	–	–	–	–	–	14.6
	PMZ2	–	–	–	6/6	5/6	4/6	0/6	–	–	1.54 × 10^6^
Oral	WT	–	–	–	3/6	1/6	0/6	0/6	–	–	9.63 × 10^3^
	PMZ2	–	–	–	–	–	6/6	6/6	6/6	6/6	> 3.05 × 10^9^

### The safety of the *P. multocida* mutant

**Figure 3:**
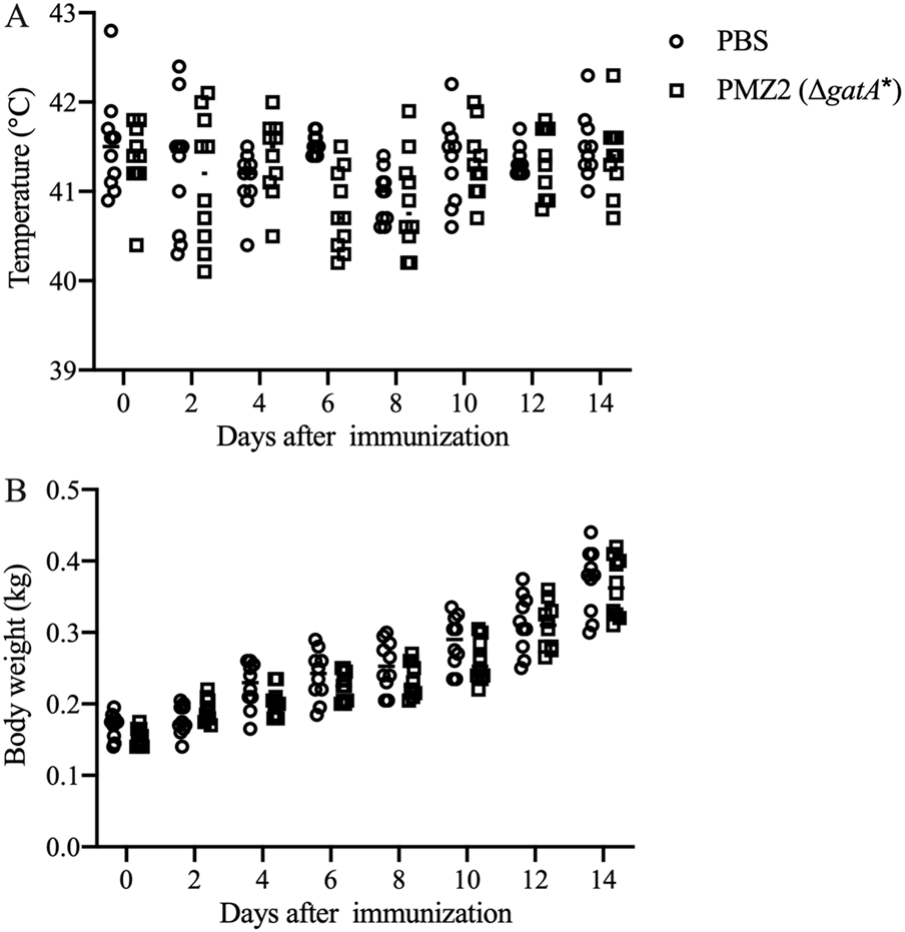
**Safety assay.** Approximately 10^9^ CFU of the mutant PMZ2 (ΔgatA^*^) or PBS was inoculated into ducklings (*n* = 10/group) orally, and then the body temperatures (**A**) and body weights (**B**) of ducks in each group were measured every 2 days during a 14-day period.

Ducks infected with a high dose of PMZ2 exhibited similar body temperatures and a comparable growth trend of body weight with animals in the PBS group (Figures 3A and B). Additionally, PMZ2-infected ducks showed no clinical symptoms of FC post-infection except depression in the first two days.

### Antibody response and serum bactericidal effect after immunization with the *P. multocida* mutant

**Figure 4:**
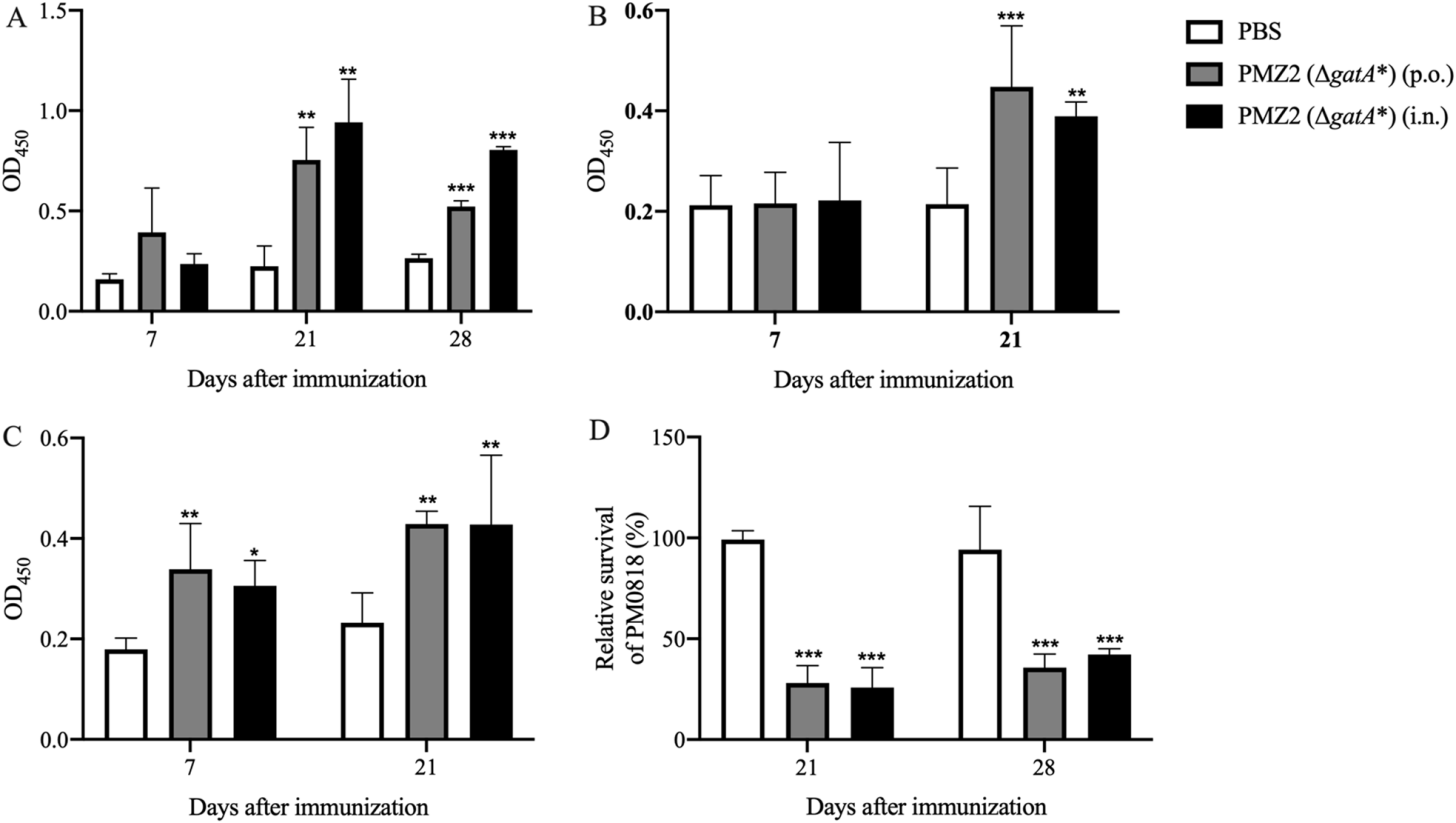
**Antibody responses and serum bactericidal assay.** Ducks were immunized with two doses of 10^8^ CFU of PMZ2 (ΔgatA*) orally or intranasally, given 2 weeks apart. PBS was also inoculated in the same way. Then, the levels of serum IgG (**A**), bile IgA (**B**) and tracheal IgA (**C**) in each group (*n* = 5) against the *P. multocida* antigens were measured by indirect ELISA at the indicated timepoints post-first immunization. Additionally, the *P. multocida* WT strain was treated with inactivated or active serum from day 21 and day 28 post-first immunization from each group for 5 h at 37 °C, and then the bacterial survival was calculated as the CFU after active serum treatment divided by the CFU after inactivated serum treatment (**D**).

There was no significant specific serum IgG production in the PMZ2 oral or intranasal group at day 7 post-first immunization, whereas at day 21 and day 28 post-first immunization, significantly increased IgG levels were induced in the two PMZ2 immunization groups compared with the PBS group (Figure 4A. Day 21, *p* < 0.01; Day 28, *p* < 0.001). Also, immunization with PMZ2 via either the oral or intranasal route triggered significant IgA responses in the bile at day 21 post-immunization (Figure 4B. Oral group, *p* < 0.001; intranasal group, *p* < 0.01) and in the trachea both at day 7 (Oral group, *p* < 0.01; intranasal group, *p* < 0.05) and day 21 (*p* < 0.01) post-immunization (Figure 4C). Additionally, after treatment with the serum from day 21 and day 28 from each group, the bacterial survival of the PMZ2 oral and intranasal groups was significantly lower than that of the PBS group (Figure 4D,  *p* < 0.001), indicating that the serum from the two PMZ2 groups elicited potent bactericidal effects against the WT strain.

### Protection efficacy provided by the *P. multocida* mutant

**Figure 5:**
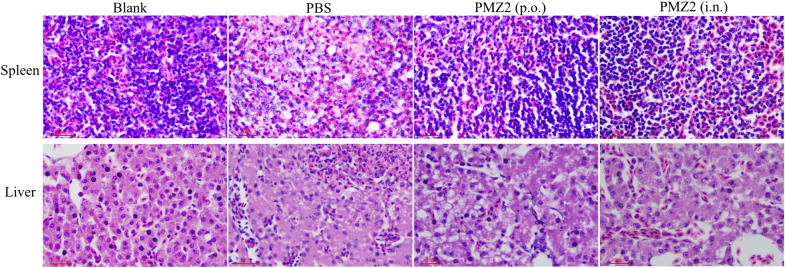
**Pathological changes in ducks.** The ducks in the immunization groups were challenged with 100-fold LD_50_ of the *P. multocida* WT strain intramuscularly 14 days post-second immunization. Then, histopathological lesions in the spleen and liver (n = 4/group) were analysed by HE staining at 24 h post-challenge. The representative results of each group were shown.

**Figure 6:**
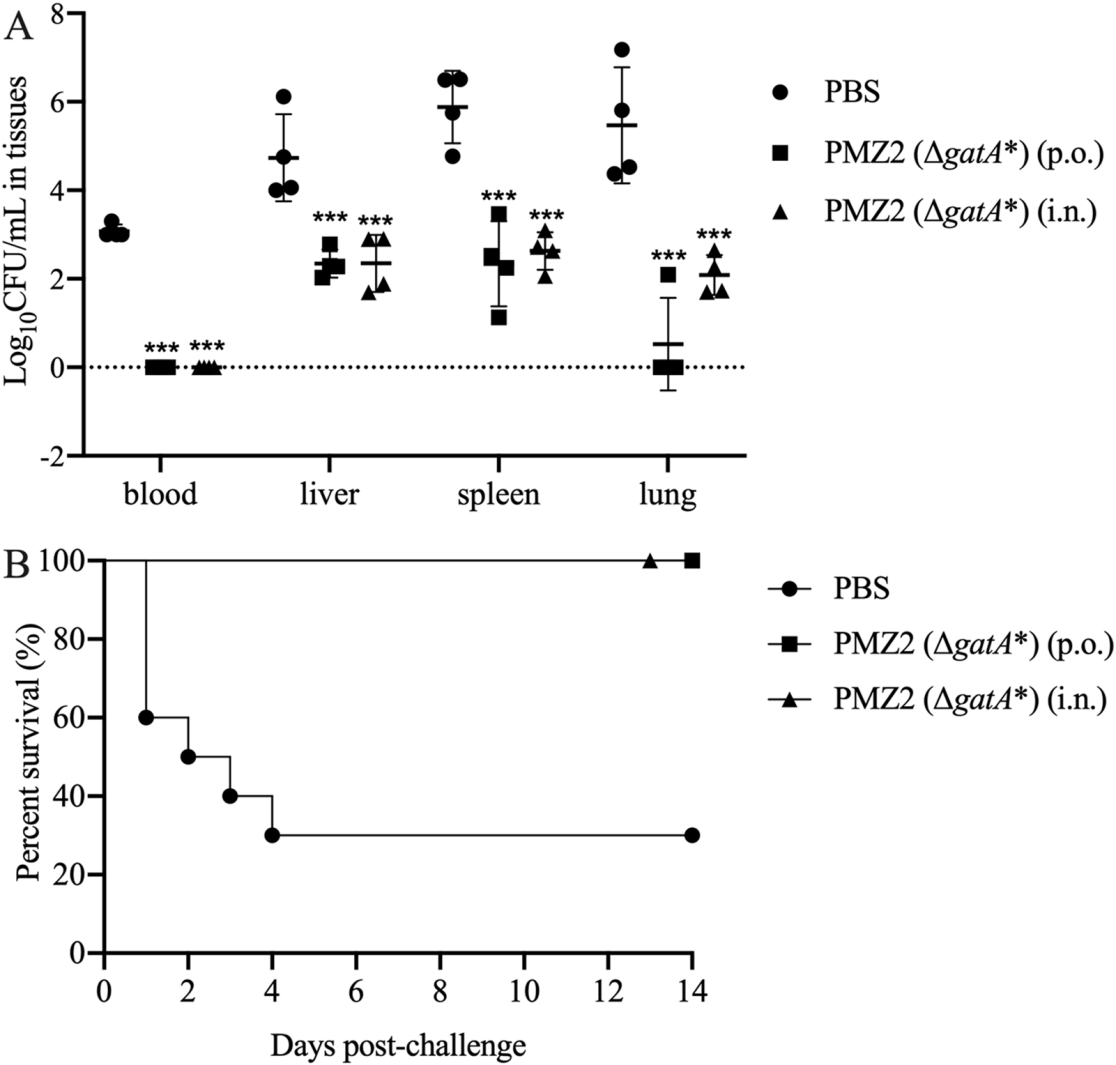
**Bacterial loads and animal survival post-challenge.** **A** The bacterial loads of each immunization group (*n* = 4/group) in the blood, liver, spleen and lung were determined as CFU/mL or CFU/g at 24 h post-challenge. **B** Animal mortality (*n* = 10/group) was also recorded daily post-challenge within a 14-day period.

The histopathological analysis post-challenge shows that ducks in the PBS group had severe lesions in the spleen and liver with structural disorder. The splenocytes exhibit severe necrosis and nuclear karyolysis and plenty of heterophils infiltrated in the spleen. Many hepatocytes show severe hydropic degeneration and necrosis with nucleus pyknosis or nuclear karyolysis in the necrotic foci; also, there was moderate congestion and infiltration of a number of heterophils in the sinusoid. By contrast, the PMZ2 oral or intranasal group display no histopathological lesions in the spleen, while some hepatocytes show severe hydropic degeneration in the oral group and mild granular degeneration in the intranasal group. The blank group displayed regular tissue structures without histopathological lesions (Figure 5). The bacterial loads of the PMZ2 oral and intranasal groups were significantly lower than that of the PBS group in the blood, spleen, liver and lungs post-challenge (Figure 6A,  *p* < 0.001). In particular, no bacteria were detected in the blood of the two PMZ2 immunization groups (Figure 6A). Furthermore, in the face of lethal challenge, 70% of ducks in the PBS group succumbed to the challenge within 4 days, while all ducks of the two PMZ2 groups survived the challenge (Figure 6B). Thus, immunization with the *P. multocida* mutant, orally or intranasally, could provide a high level of protection against lethal challenge with the homologous strain.

## Discussion

LPS, the major cell wall component of the bacterium, plays an important role in disease progression in chickens [30]. Mutations of certain synthesis genes of the LPS inner core or outer core in the *P. multocida* WT strain led to significant attenuation of bacterial virulence in chickens [21, 22, 31]. Our recent study also demonstrated the essential roles of the outer core glycotransferase genes *pcgD* and *hptE* in bacterial colonization and virulence in ducks and confirmed the greater impact of *pcgD* on bacterial pathogenesis than *hptE* [23], which was somewhat contradictory to previous studies conducted in chickens [21, 22]. The inconsistent findings indicated the necessity of studying LPS gene functions in different avian species. This study found that deletion of another outer core glycotransferase gene *gatA* and the first four bases of the *hptE* gene gave rise to a further reduction in bacterial virulence compared with the *pcgD* mutation. The loss of the start codon was very likely to influence the expression of functional HptE, since complementation with only the *gatA* gene in the resulting mutant strain PMZ2 did not fully restore the wild-type LPS phenotype. Nevertheless, it seems impossible that the *hptE* mutation made a great contribution to the striking virulence attenuation of PMZ2, which had a 10^5^-fold higher intramuscular LD_50_ and significantly decreased colonization in tissues than the WT strain, because it neither decreases bacterial loads in tissues nor reduces virulence upon intramuscular infection [23]. We predicted that the *gatA* deletion was fully or at least largely responsible for the virulence attenuation of PMZ2, which needs to be proven by the construction of a precise *gatA* gene mutant in future studies.

The remarkably reduced virulence and the ability to maintain a certain level of bacterial loads in tissues possessed by PMZ2 prompted us to evaluate its potential as a qualified live vaccine in ducks. The mutant was inoculated into ducklings via the oral or intranasal route for detection of immunogenicity and protection efficacy. It was found that both routes of two-dose immunizations induced potent systemic and mucosal antibody responses and a high level of protection against lethal challenge, suggesting that PMZ2 could penetrate the mucosal barriers in the respiratory tract and intestine and disseminate to peripheral lymphoid tissues to stimulate adaptive immunity. The bacterial translocation from mucosal sites to other organs has been observed in turkey, chicken, buffalo and cattle [32–34]. LPS is released into the blood during the early stages of *P. multocida* infection and destroys the blood endothelial cells indirectly [33], which may increase the permeability of the blood vessels and then facilitate bacterial migration. Also, mucosal macrophages were implicated in bacterial trafficking [2]. Additionally, the production of specific bile IgA in the PMZ2 intranasal group or tracheal IgA in the oral group confirmed the induction of common mucosal immunity [35] by PMZ2 vaccination in ducks. The prevention of natural infection by virulent bacteria is an essential property for effective vaccines, and circumstantial evidence points towards the respiratory tract as the main site of entry for *P. multocida* infection in birds [2]. The existence of common mucosal immunity in ducks provided the basis for the effectiveness of oral immunization with live *P. multocida* vaccines.

In the face of lethal intramuscular challenge, the immunized ducks show decreased bacterial loads, alleviated tissue lesions and 100% survival, indicating that immunization with PMZ2 carrying an incomplete LPS structure stimulates protective adaptive immunity independent of the LPS outer core-stimulated immune responses that are required for the potency of *P. multocida* killed whole-cell vaccines [17, 36]. This finding was consistent with a previous study showing that *P. multocida* L1 live-attenuated strains expressing either full-length or truncated LPS displayed comparable protection against the isogenic parent strain [17]. A similar phenomenon was also found in *Salmonella* studies, suggesting that the protective role of antibodies specific to the LPS is not indispensable for live attenuated *S.* Typhimurium vaccines [29, 37]. Some membrane proteins of *P. multocida*, including PlpE, Cp39, OmpH, PtfA and FHAB2, have been proven to be protective antigens that stimulate protective immunity against FC [38–42]. It would be of interest to examine the role and contribution of these antigens in the induction of protective immunity by live attenuated *P. multocida* vaccines.

The global epidemiology of FC is complex with diverse serovars mainly Heddleston serotypes 1, 3, 4 in different regions worldwide [4, 43–45], which poses a substantial challenge for current commercial FC vaccines especially inactivated bacterins that were designed based on single serotype [14]. Live attenuated vaccines have been proven to confer a certain level of cross protection [17], therefore cross-immunity potential of the PMZ2 needs to be evaluated in further studies. Additionally, to ensure the complete safety of live vaccines, it is generally required to include multiple virulence genes for targeted mutations to decrease the probability of virulence reversion. Unfortunately, although some genes, such as *fis*, *hfq* and *hbpA*, are highly virulence correlated [46–48], few of them have been targeted for the evaluation of vaccine potential. Therefore, screening for more suitable virulence genes, either proven or newly discovered, that meet the characteristics of effective live vaccines, and introducing one or more gene mutations into PMZ2 to ensure complete safety still need to be conducted in later studies.

In summary, this study constructed a novel live attenuated *P. multocida* vaccine strain PMZ2 with deletion of the entire *gatA* gene and part of the *hptE* gene that produced a truncated LPS structure. A high dose of oral inoculation with PMZ2 did not cause adverse effects on the body temperature and body weight gain of ducks, and immunization with the mutant orally or intranasally induced a high level of serum IgG with strong bactericidal effects and significant mucosal IgA responses. Furthermore, immunized ducks in the PMZ2 oral and intranasal groups exhibited significantly reduced bacterial loads in the blood, spleen, liver and lung, alleviated spleen and liver lesions and 100% survival against lethal challenge with the WT strain. Therefore, the newly constructed *P. multocida* PMZ2 strain was highly attenuated while maintaining good immunogenicity, and immunization via the oral or intranasal route provided a high level of protection efficacy against FC in ducks.

## Supplementary Information

**Additional file 1. Primers used in this study.**

**Additional file 2.** **Characterization of the *P. multocida* mutant strain via PCR.** The WT strain and PMZ2 (ΔgatA^*^) mutant were identified using primers MIA-1F/P1-R, P2-F/MIA-2R, and MIA-3F/3R to confirm the gene mutation. M refers to the DNA marker.

## Abbreviations

*P. multocida*: *Pasteurella multocida*
FC: fowl cholera
LPS: lipopolysaccharide
CU: Clemson University
Hep: heptose
Gal: galactose
LD_50_: median lethal dose
WT: wild-type
BHI: brain heart infusion
*E. coli*: *Escherichia coli*
LB: Luria–Bertani
TSA: tryptic soybean agar
*kanR*: kanamycin resistance
CFU: colony forming unit
ELISA: enzyme-linked immunosorbent assay
BSA: bovine serum albumin
HRP: horseradish peroxidase
HE: haematoxylin and eosin
