# Supplementary material for: Immunogenicity and protection of a Pasteurella multocida strain with a truncated lipopolysaccharide outer core in ducks
Source: Vet Res. 2022 Mar 2;53:17. doi: 10.1186/s13567-022-01035-y (PMC8889768; doi:10.1186/s13567-022-01035-y)
Supplement: Supplementary file 1 — Additional file 1. Primers used in this study. [file 13567_2022_1035_MOESM1_ESM.docx]

**Additional file 1. Primers used in this study.**

| Primer | Sequence 5’-3’ |
| --- | --- |
| D*gatA^*^*-1F | CGGGGTACCATCACTTCGGTGATAGCTTTG |
| D*gatA^*^*-1R | ccgttgaatatggctcatatattctcctaatttttatg |
| D*gatA^*^*-2F | gctcgatgagtttttctaataaaaagcatgctacaa |
| D*gatA^*^*-2R | CCCCCGGGGGGTGAAATTGTCCCTCCAG |
| *kanR*-*gatA^*^*-F | cataaaaattaggagaatatatgagccatattcaacgg |
| *kanR*-*gatA^*^*-R | ttgtagcatgctttttattagaaaaactcatcgagc |
| C*gatA*-F | CCGGTACCATGAAATTACCTAAAATTATAGTAATTAG |
| C*gatA*-R | ATTTGCGGCCGCTCATTTCAAACCCGCTCTTCTCTC |
| C*gatA**-hptE*-F | GGGGTACCGGAGAATATATGAAATTACC |
| C*gatA-hptE* -R | TTGCGGCCGCCTATTTGACAAGCCAT |
| *tpiA*-F | CCGCTCGAGgcgaaagacgatattgctc |
| *tpiA*-R | CCGGAATTCaattttctccgtattaaag |
| MIA-1F | CCTTATCGAAGCAGGAATTGATG |
| MIA-2R | TCGACCGCACTTTGCTCATC |
| MIA-3F | AATTGGTTGCGCTATGAGCC |
| MIA-3R | GCTAGGCTCGATTCCGTAG |
