# Supplementary figures and images for: Immunogenicity and protection of a Pasteurella multocida strain with a truncated lipopolysaccharide outer core in ducks
Source: Vet Res. 2022 Mar 2;53:17. doi: 10.1186/s13567-022-01035-y (PMC8889768; doi:10.1186/s13567-022-01035-y)

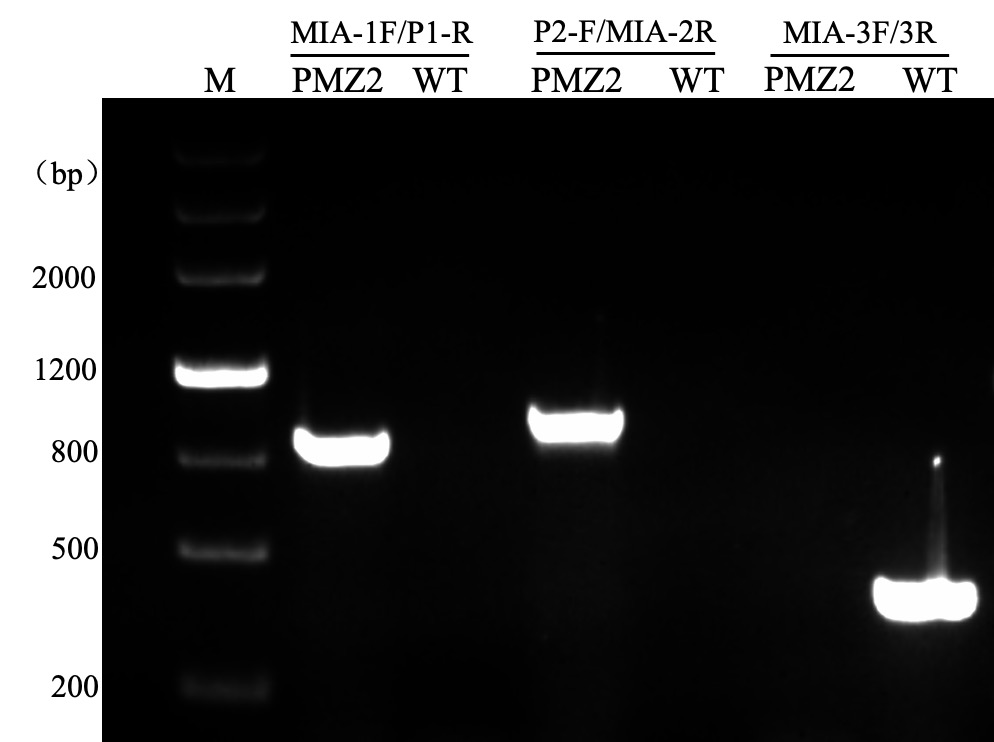

Supplement: Supplementary file 2 — Additional file 2. Characterization of the P. multocida mutant strain via PCR. The WT strain and PMZ2 (ΔgatA*) mutant were identified using primers MIA-1F/P1-R, P2-F/MIA-2R, and MIA-3F/3R to confirm the gene mutation. M refers to the DNA marker. [file 13567_2022_1035_MOESM2_ESM.jpg]
